# GMOseek: a user friendly tool for optimized GMO testing

**DOI:** 10.1186/1471-2105-15-258

**Published:** 2014-08-01

**Authors:** Dany Morisset, Petra Kralj Novak, Darko Zupanič, Kristina Gruden, Nada Lavrač, Jana Žel

**Affiliations:** Department of Biotechnology and Systems Biology, National Institute of Biology, Večna pot 111, SI-1000 Ljubljana, Slovenia; Department of Knowledge Technologies, Jožef Stefan Institute, Jamova cesta 39, 1000 Ljubljana, Slovenia; University of Nova Gorica, Vipavska 13, 5000 Nova Gorica, Slovenia; CropDesign N.V., GBB/RY - BIO 2, Technologiepark 21C, 9052 Gent (Zwijnaarde), Belgium; DA-MA s.p., Doropolje 14A, 3225 Planina pri Sevnici, Slovenia

**Keywords:** Genetically Modified Organism, Matrix approach, Constraint optimization, Cost efficient GMO testing

## Abstract

**Background:**

With the increasing pace of new Genetically Modified Organisms (GMOs) authorized or in pipeline for commercialization worldwide, the task of the laboratories in charge to test the compliance of food, feed or seed samples with their relevant regulations became difficult and costly. Many of them have already adopted the so called "matrix approach" to rationalize the resources and efforts used to increase their efficiency within a limited budget. Most of the time, the "matrix approach" is implemented using limited information and some proprietary (if any) computational tool to efficiently use the available data.

**Results:**

The developed GMOseek software is designed to support decision making in all the phases of routine GMO laboratory testing, including the interpretation of wet-lab results. The tool makes use of a tabulated matrix of GM events and their genetic elements, of the laboratory analysis history and the available information about the sample at hand. The tool uses an optimization approach to suggest the most suited screening assays for the given sample. The practical GMOseek user interface allows the user to customize the search for a cost-efficient combination of screening assays to be employed on a given sample. It further guides the user to select appropriate analyses to determine the presence of individual GM events in the analyzed sample, and it helps taking a final decision regarding the GMO composition in the sample. GMOseek can also be used to evaluate new, previously unused GMO screening targets and to estimate the profitability of developing new GMO screening methods.

**Conclusion:**

The presented freely available software tool offers the GMO testing laboratories the possibility to select combinations of assays (e.g. quantitative real-time PCR tests) needed for their task, by allowing the expert to express his/her preferences in terms of multiplexing and cost. The utility of GMOseek is exemplified by analyzing selected food, feed and seed samples from a national reference laboratory for GMO testing and by comparing its performance to existing tools which use the matrix approach. GMOseek proves superior when tested on real samples in terms of GMO coverage and cost efficiency of its screening strategies, including its capacity of simple interpretation of the testing results.

**Electronic supplementary material:**

The online version of this article (doi:10.1186/1471-2105-15-258) contains supplementary material, which is available to authorized users.

## Background

Since the first commercialization in 1996, genetically modified organisms (GMOs) have gained significant shares in agriculture and food chains at a global scale [1, 2]. As an answer to the public concern regarding the use of plant biotechnology products, the authorization, labeling, and compliance control of GMOs is a requirement for regulations in many countries.

A GMO is an organism whose genome was modified by introducing a foreign genetic construct (a transgene) consisting of several genetic components (gene of interest, regulatory sequences for the gene to enable its function in the host organism, etc.…). Therefore, the most appropriate methods for GMO detection are based on testing the presence of the DNA sequence of a given GMO (called "event"). To date, the preferred technique to perform these tests is the Polymerase Chain Reaction (PCR) or its derivative real-time PCR [3–6]. The PCR assays can target commonly used genetic components (or groups of genetic components) found in GMOs (screening tests) [4], or they can aim at identifying the specific signature of one given GM event for identification purposes (event-specific tests) [7].

Together with the increasing number of genetically modified (GM) events commercialized and in pipeline for commercialization in the recent years, their complexity and diversity in terms of the crop taxa and genes involved have grown rapidly. As a consequence, the screening phase became more complicated with less obvious combinations of screening tests to cover a wide range of GM events. Regarding the identification phase and given the high number of GM events to be scrutinized, using only event-specific assays is not economically sustainable and is limited to the detection of known events only.

To face the challenge of maintaining the cost of GMO analysis affordable with an enhancing spectrum of candidates to be detected, the so-called "matrix approach" [8] has been adopted by numerous laboratories, i.e. the members of the European Network of GMO Laboratories (ENGL). The matrix is a tabulated dataset in which each row represents a specific GM event and the columns represent the genetic elements composing the GM event which can be used as targets for analytical test methods. The matrix approach combines the use of screening and event-specific assays [9–12]. The screening phase employs combinations of screening assays allowing a large coverage of GM events. By comparing the results of these assays with tabulated data about the theoretical presence/absence of the targeted components in individual events (the matrix), the analyst discards the GM events not detected (and therefore assumed to be absent) in the tested sample. Event-specific assays are then used in the subsequent analytic phase for identifying the GM event(s) present in the sample. In the case that the identified GM event(s) does (do) not match with the screening results pattern, further analysis has to be performed to elucidate the origin of the unexplained positive signals [5, 12–14]. The advantage of the matrix approach is the reduced number of PCR tests needed to achieve the identification of the GM event(s) present in the sample and/or to conclude on the compliance of the tested sample.

Two key elements lead to the correct use of the matrix approach: availability of information regarding the genetic components in the individual GM events, and correct use of this information and results of tests to achieve proper conclusions regarding the sample compliance with the GMO regulations.

Since its first introduction within the European FP5 GMOchips project [15], the implementation of the matrix approach was limited due to the dispersion and lack of completeness of the information regarding the GMO genetic components found in several databases, which are mainly dedicated to GMO risk assessment [16–21]. Several individual efforts were made to demonstrate the use of the matrix approach in GMO analysis [10–12, 22, 23]. Recently, a coordinated effort under the framework of the European ERA-NET GMOseek project led to the compilation of the most comprehensive set of data dedicated to the implementation of the matrix approach. This set of data has since been made available [8].

The matrix approach has three main steps: 1) selection of the screening assays to be used in the first analytical phase, 2) comparison of the screening results with the tabulated data to decide on the next, identification phase (using event-specific assays), 3) the interpretation of both the screening and identification phase patterns to correctly conclude on the sample composition in terms of GM events.

Just a few matrix approach tools are available [8, 11, 12, 22, 23]. These tools are often not amenable for wide use of the GMO testing community because of their lack of flexibility and availability. The GMOfinder tool [23] is not publicly available because of intellectual property issues, the COSYPS system [11] is limited to the SYBR®green PCR chemistry, and Excel applications [12] enable only low combinatory approach to support decision making in GMO detection. Finally, none of these tools considers the cost-efficiency of GMO testing, as they only focus on the identification of the events in the sample.

In a previous study, we have developed the GMOtrack tool that finds cost-efficient two-phase (screening–identification) sample-centered testing strategies [22]. While that study reports on major cost benefits of using the sample-centered cost-optimization approach to GMO testing, the adoption of GMOtrack in routine laboratories was limited by two factors. One major shortcoming of GMOtrack is limited user unfriendliness and support only for the first step of the matrix approach - the selection of screening assays, omitting the selection of necessary event-specific assays to be performed based on screening results, and also omitting the support for the interpretation of the results from both screening and identification phase to conclude the analysis. The second issue is that the algorithm used in GMOtrack performs exhaustive search for finding optimal two-phase testing strategies.

When GMOtrack was developed in year 2008, an exhaustive approach was feasible, as then only 22 GM events needed to be tested in the EU. However, it is not feasible to use it with large datasets such as the recent GMOseek matrix [8], given that more than 50 EU-approved GM events, and more than 320 GM events and 240 different genetic elements are listed globally.

The main purpose of this study is to present and evaluate the GMOseek software tool that upgrades the GMOtrack tool in several ways. GMOseek uses an improved search strategy which quickly finds near optimum cost-efficient two-phase sample-centered testing strategies within large datasets like the matrix described in [8], utilizing a conventional laboratory computer. GMOseek also provides a user-friendly interface with a decision support system, which guides the user through all the three steps of the cost-efficient matrix GMO testing approach: from the selection of screening assays, deciding on the event-specific assays to be performed and the final interpretation of the results. The GMOseek tool was evaluated at the National Reference Laboratory for GMO testing food and feed (the "TestLab" in the following), demonstrating its capacity to ease the analyst task and reduce the total analysis costs.

The advantages and limitations of the matrix-based approach have been discussed in recent publications, including the issues of the assay sensitivity and specificity that can be responsible for interpretation errors [8, 9, 13]. The reader is invited to consult these publications for further considerations regarding assay performance when using this approach.

## Implementation

GMOseek is a user-friendly software tool with a decision support system which guides the user through the three steps of the cost-efficient sample-centered matrix GMO testing. The software is developed in Java and runs on any system with a Java Virtual Machine 1.5 or later. It is packed and deployed as one file which is named *GMO.jar*. The program can be freely downloaded from the web page http://kt.ijs.si/software/GMOtrack/GMOseek.html or http://www.gmoseek.com/gmoseek placed on a user’s computer and run by a double click on the file from a file manager or by java -jar GMOseek.jar from the command line.

The matrix approach for GMO traceability relies on a matrix of GM events relevant to the food, feed and seed legislation and their genetic components. The sample-centered cost-efficient GMO testing approach used by GMOseek can additionally incorporate the data about laboratory analysis history in the form of probability of GMO presence and information about the sample to be tested. GMOseek can easily adapt to new situations on the market by changing the input data matrix, which includes the data about GMOs, methods (assays) for detecting GMOs and probabilities of GMO presence. The data format of GMOseek is compatible with the GMOtrack data format. The data can be downloaded from the project’s website ( http://www.gmoseek.com/gmoseek), where the comprehensive matrix from Block and collaborators [8] and another matrix with data about GM events approved in the EU as well as the events regulated under EC 619/2011 [24] are available to be used directly with the GMOseek program. These datasets can be further tailored to a situation at hand by editing in a spreadsheet program (like OpenOffice Calc or MS Excel) and save to a tab-separated file.

In the sample-centered approach to analytical GMO tracking, the testing strategy is tuned to the given sample in order to minimize the total analysis cost, instead of using the same testing strategy for all samples. Sample-centered testing strategies generated by GMOseek have a screening and an identification phase. To optimize the total analysis cost, the sample-centered approach finds a combination of screening assays that best trades off the screening and the expected event-specific costs. The expected event-specific cost is estimated from the data in the matrix, laboratory analysis history and prior knowledge about the sample (e.g. is it a food or feed sample). The GMOseek testing strategies are in-line with the guidelines for the preparation of GMO screening analysis using the matrix-based approach as described by Kralj Novak *et al.* [22].

The GMOseek system for guiding the analyst through the three steps of the cost-efficient matrix GMO testing approach has two main components: the GMOseek algorithm for computing near-optimum two-phase sample-centered GMO testing strategies and a decision support system for guiding the analysis and interpreting the results. The remainder of this section describes the two main components.

### The GMOseek algorithm

The task addressed by the GMOseek algorithm can be formulated as follows: given a matrix of GM events and available screening assays, prior probabilities of GMO presence (estimated from historical data) and information about the species of the sample at hand, find a two-phase testing strategy with the lowest total expected cost. The total expected cost of a two-phase testing strategy is the sum of the screening cost and the expected event-specific cost, where the expected event-specific cost is computed from the probabilities of GMO presence. The strategies either identify the GMO present in the sample by an event-specific assay or confirm its absence by either a screening or an event-specific assay for all the GMs in the dataset.

For more details on probability calculation and cost estimation, see the GMOtrack formal background [22] and supplementary material available at http://kt.ijs.si/software/GMOtrack/. As previously described [22], the cost of one run of PCR assays for the chosen laboratory is a linear function of the number of assays (numAssays) according to the equation g(numAssays) = 21.18 · numAssays + 91.82. It is a simplification of the real situation with a relative absolute error of 3 %. The cost of one run takes into account both the labor and the material / reagent costs. The GMOseek cost computation formula is the same, but algorithmically improved by first computing the parts of the cost that contribute the most. In this way, the cost computation can be stopped when the partial cost exceeds the best cost so far, leading to major computation time savings.

The GMOseek algorithm uses a smart searching strategy for finding the optimal GMO testing strategy. It considers the assays that contribute the most to the coverage of the whole GMO set first in order to quickly generate a good (in terms of total expected cost) combination of assays. It then generates the other combinations of assays, which are pruned if their cost is higher than the best cost so far. As the cost computation is very complex, it is interrupted if the partial cost exceeds the best cost so far. The algorithm prunes a set of candidate solutions if the screening phase cost of new candidate strategies is higher than the total expected cost of the best solution so far. The algorithm stops when all the candidate screening assays are either evaluated or pruned.

The best solution so far, its total expected cost and its coverage are printed on the interface when generated. The user can interrupt the search at any time, if he/she is satisfied with the proposed solution, or continue the search to the end, waiting for the best solution to be proved.

### The GMOseek decision support system

GMOseek has an interface for entering the information about the sample at hand and choosing the matrix with available assays and estimated probabilities of GMO presence. This information is used by the GMOseek algorithm to tailor the screening to the given sample. The combination of screening assays proposed by the GMOseek algorithm can be used in wet-lab or, alternatively changed and other screening can be performed.

GMOseek has an interface for entering (clicking) screening wet-lab results. It compares the screening results with the matrix data on the fly and points out which event-specific assays need to be performed. Finally, the result of event-specific testing can be entered and the system interprets the results and checks for inconsistencies:

- Positive event-specific tests prove the presence of respective GM events
- A positive screening assay suggests a positive GM event, but event-specific results can contradict this
- GMOseek warns for potential stacked genes when two or more events of the same species are identified

Finally, GMOseek can also be used to select new GMO screening targets and estimate the profitability of developing new GMO screening methods. GMOseek can be used to see which and if and in which scenario (e.g. change of GMO frequencies, introduction of so far not authorized GM events) the potential new screening assays would be used in the optimal testing strategy. These results should be compared with the cost of testing with only the existing screening assays, also calculated by GMOseek. The difference in costs in different scenarios is a good estimate of the profitability of candidates for new screening assays.

It should be emphasized that all the functionalities of GMOseek can be used together or separately, as the user prefers. In many laboratories, users prefer to use a fixed set of screening assays of their choice. In such a case, they can still largely benefit from the GMOseek decision support system for selecting the necessary event-specific assays that need to be performed based on the screening results and for interpreting the results of both the screening and event-specific testing phase.

## Results and discussion

This section describes the functionality of the GMOseek software together with performance and functionality comparisons with its predecessor, the GMOtrack software. There is also a discussion of the intended use of the software, and the benefits that are envisioned together with an outline for the planned future development of new features.

### From GMOtrack to GMOseek

GMOseek is the successor of GMOtrack, and its development was motivated by the successful applications of GMOtrack and by the fact that the matrix approach became the most obvious strategy for contemporary GMO testing. GMOtrack was the first system addressing the routine laboratory-level GMO tracking as a cost optimization problem. The search procedure of GMOtrack is exhaustive; this means that the algorithm generates all the possible combinations of up to *m* screening assays and selects the one with the lowest expected cost for a sample at hand. Its applications show major cost benefits of shifting from "the same strategy for all samples" to "sample-centered GMO testing strategies". GMOtrack was developed at the level of "proof-of-concept", hence its search procedure for finding the optimal combination of assays for testing a sample at hand was not optimized. Moreover, GMOtrack has a command-line utility that only suggests the optimal set of screening assays to be applied in the screening phase and does not support the interpretation of wet-lab results. Nevertheless, all the definitions, strategies and other research background defined and developed within GMOtrack have been incorporated in GMOseek, which overcomes its predecessor’s deficiencies and provides new functionalities.

**Figure 1:**
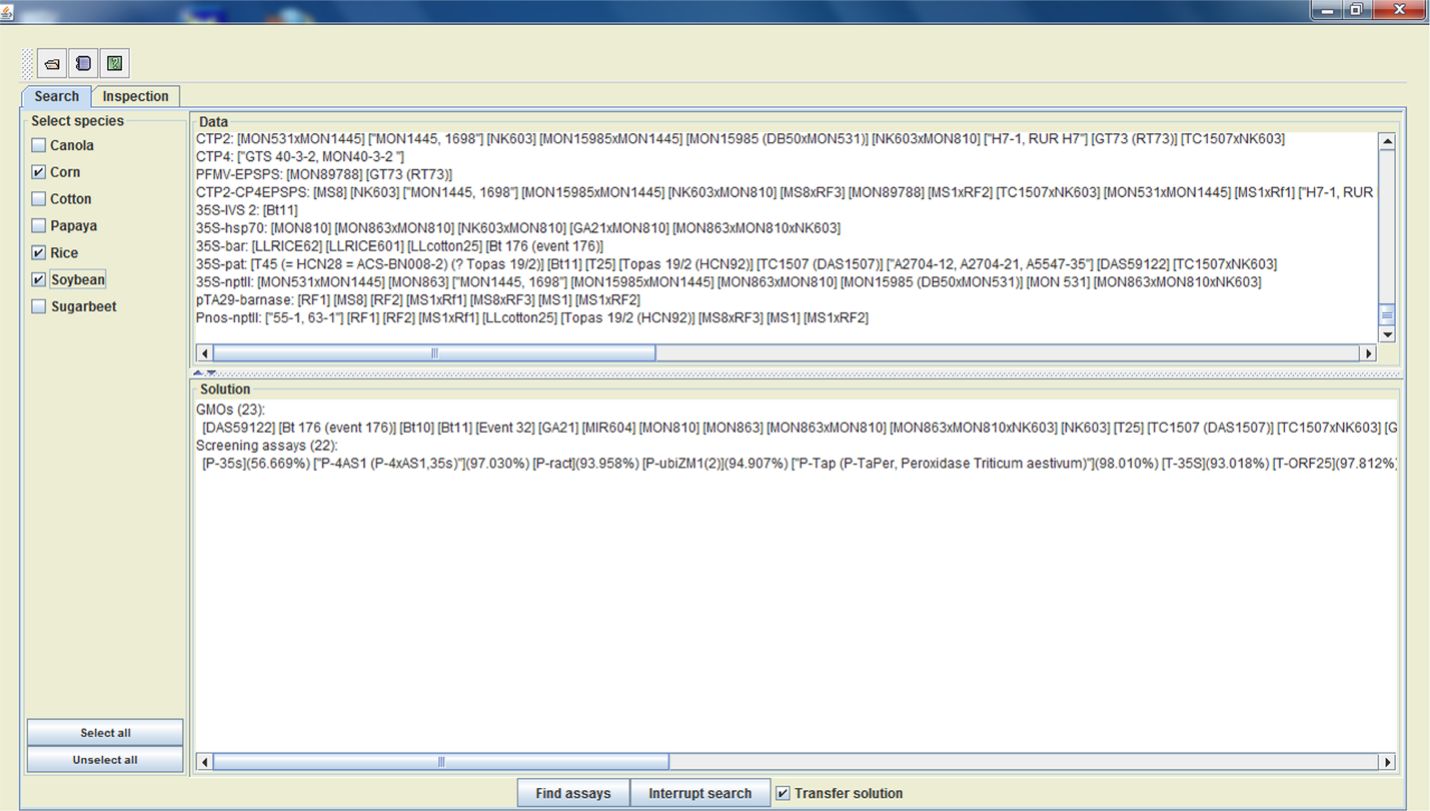
**Search platform of the GMOseek software (part 1).** Upper toolbar, left command button: Command enabling to browse, select and load a dataset. Upper toolbar, central command button: Command enabling to open a table containing a dataset that was last used. Upper toolbar, right command button: Command opening the help window containing the user manual. Left panel: Species present in the data set. The user can choose to select all the species or choose the ones of interest (e.g. the ingredient of the sample) for the search. Upper, middle panel: contains information about the definition. The information is about chances of a GMO appearing in a sample and about a GMO detection of a screening assay. Lower, middle panel: Once plant species are selected, displays a list of possible GM events and a list of screening assays which can detect these GM events. A probability of a negative result of a test is attached to each screening assay (in a case that the screening assay would be used alone). Lower tool bar, left command button: Command enabling the search start "find assay". Lower tool bar, right command button: Command enabling manual interruption of the search. Lower tool bar, selection field: if marked, this selection allows transferring the search results (best solution) to the inspection platform. Information bar (bottom): Information about the path to the loaded dataset.

**Figure 2:**
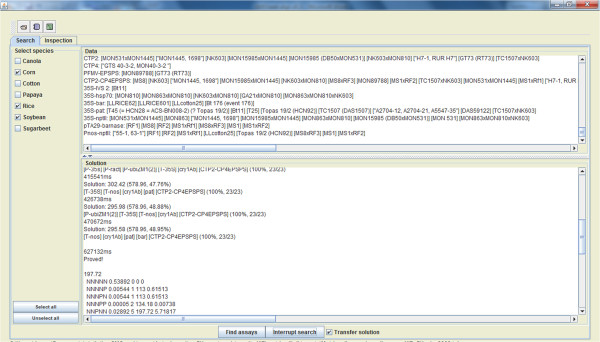
**Search platform of the GMOseek software (part 2).** Lower, middle panel: During the search process, this panel lists the combinations of assays found as follows: time (in ms) the solution is found, expected cost of the solution in arbitrary unit. In brackets are indicated the cost if only event-specific assay would be used, and the savings of the proposed solution in comparison with this "all event-specific strategy".genetic components to be targeted. In bracket is indicated the coverage of this screening solution in percentage, and as the ratio of the covered GM events vs. the total GM events in the dataset.

**Figure 3:**
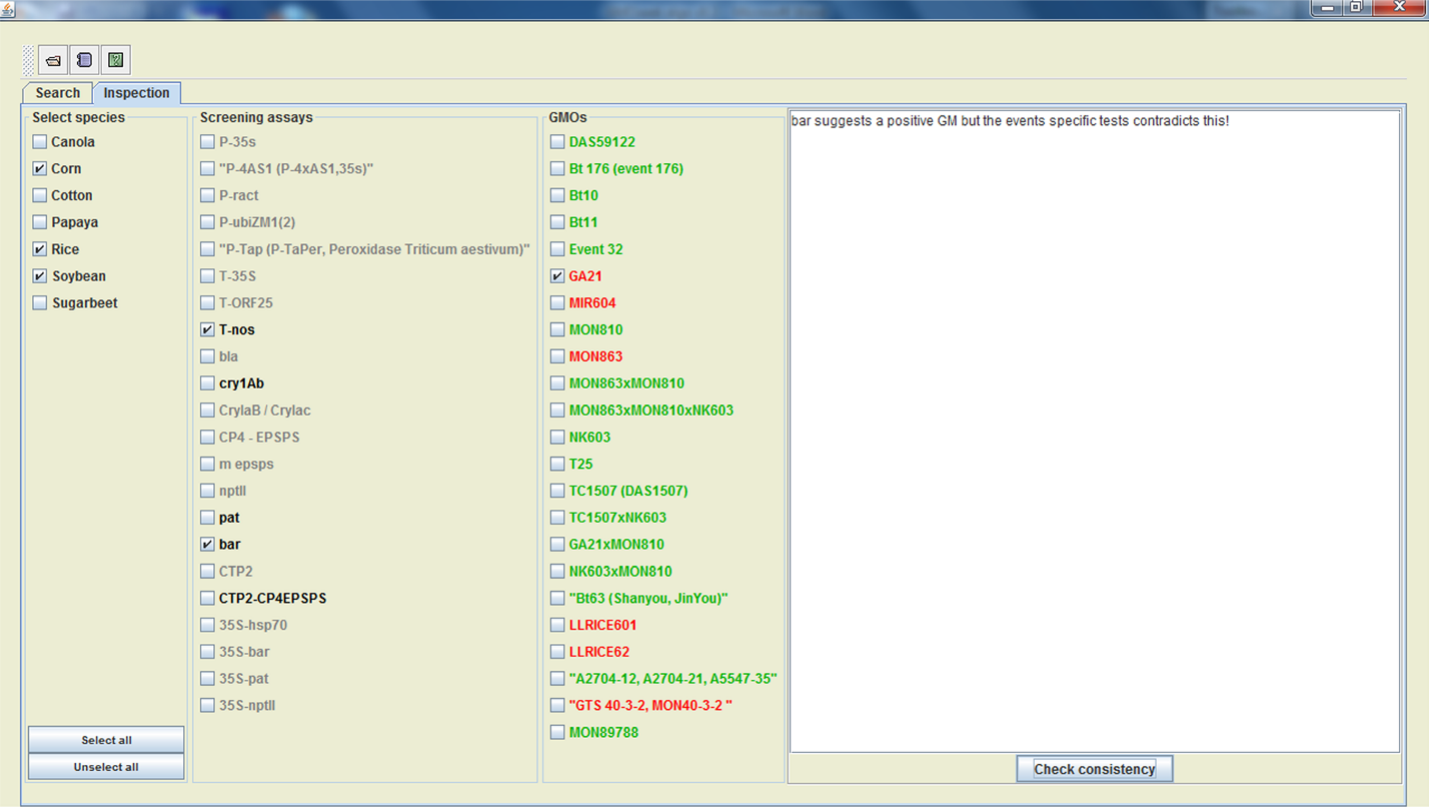
**Inspection platform (Decision Support System) of the GMOseek software.** Extreme left panel: Species present in the data set. The user can choose to select all the species or choose the ones of interest (e.g. the ingredient of the sample) for the inspection.Middle left panel: Screening assays that can be performed for the loaded dataset. The screening assays indicated in black are the ones chosen for the experimental screening phase. The grey assays are not tested. The user can select the assays to be tested by right clicking on each screening assay. Left clicking a screening assay indicated the positive outcome of a screening assay (un-ticked screening assays result in negative outcome). Middle right panel: Event-specific assays that can be performed for the loaded dataset. Depending on the outcomes of the screening phase, GM events appear green (not present in the sample) or red (possibly present in the sample, to be tested). After the experimental identification phase, the user indicates the outcome of the event-specific assays, a ticked GMO meaning a positive result of the event-specific assay for this GMO. Extreme right panel: consistency panel. After clicking on the command button "check consistency", a message appears indicating the absence of consistency between the screening phase and identification phases, or eventually the possible inconsistency of the experimental results.

Compared to GMOtrack, the GMOseek algorithm uses a constraint optimization paradigm to limit the search space when searching for the optimal combination of screening assays to get the optimal testing strategy. By using this approach, large parts of the search space can be pruned, which leads to a much faster computation. GMOseek has a much lower time complexity compared to GMOtrack, making it feasible to generate testing strategies comprising combinations of tens of screening assays selected within a pool of hundreds of potential screening assays. When used on large datasets, GMOseek can be stopped before reaching the optimal solution; in such cases, it does not guarantee to find the optimal testing strategy. Another major change compared to GMOtrack is that there is no objective set (constraints) in the search option in terms of minimum coverage of GM events during the screening phase, nor the maximum number of screening events.We developed the GMOseek system which incorporates the GMOseek algorithm and a user friendly interface that is designed to support the following steps of the routine GMO testing laboratory. First, the dataset which can be tuned to the sample at hand is loaded (Figure 1). In the data, the probability of appearance of a GMO incorporates the background knowledge about the sample (Figure 1). The user chooses the crop species present in the sample (included also in the dataset loaded). After activating the search mode, the GMOseek system then generates the near-optimal testing strategy for the sample (Figure 2). The user can decide not to wait until the GMOseek system finds a near-optimal solution by manually interrupting the search and using the proposed assay combination found so far.On the inspection panel, the user can visualize the combination of assays obtained during the search phase. This combination can be completely amended at hand by the user. When the actual experimental screening is completed following the combination chosen by the user, the screening results (positive/negative outcomes) are manually entered into the GMOseek system (Figure 3). GMOseek then suggests which event-specific assays need to be done to identify the presence of all possible GM events in the sample (Figure 3). After the experimental identification phase is completed, the event-specific test results are entered and the GMOseek system interprets them and provides warning for possible stacked genes and for inconsistent results (Figure 3).

The GMOseek tool can be used for many purposes. The major expected use is for routine laboratory testing, to choose the best combination of screening assays for a sample at hand (food, feed, and seeds). Another purpose of GMOseek is to guide the analyst through the whole process until the decision making regarding the sample compliance. For this, the analyst can decide to use the combinations of screening assays proposed by the system or amend it. The inspection platform is independent from the search platform. Therefore, the user can also decide to use GMOseek only to interpret the wet-lab results. Finally, another possible use of GMOseek is to estimate the profitability of developing new screening assays, based on the new GM events available on the (global) market and estimates of their occurrences on the market. As it was already the case with GMOtrack, the simple format of the dataset (tabulated matrix) allows the user to tailor his search with GMOseek based on his/her own needs.

### The usability of GMOseek tested on different datasets with increasing sizes (Table 1) is shown in Table 2

**Table 1:** **Size of the datasets used to compare GMOseek and GMOtrack performance**

Dataset name	Number of genetic components	Number of GM events	Combinations to be computed
**GMO_EU_2005.tab**	24	20	55,454
**GMO_EU_2008.tab**	25	22	68,405
**GMO_EU_2010.tab**	85	55	102,425
**GMO_EU_2012.tab**	121	76	295,361
**GMO_all_2012.tab**	220	247	1,774,850

*Number of genetic components: number of components to be considered for the screening phase assays*.

*Number of GM events: number of GM events to be covered in the dataset*.

*Combinations to be computed: number of combinations to be computed to generate screening sets, according to GMOtrack.*

**Table 2:** **Comparison of GMOtrack and GMOseek screening strategies**

Dataset name	Expected cost (event-specific assays only)	Lowest analytical cost GMOtrack	Combination GMOtrack (number assays)	Coverage GMOtrack (%)	Lowest analytical cost GMOseek	Combination GMOseek (number assays)	Coverage GMOseek (%)
**GMO_EU_2005.tab**	515.42	234.24	4	100	234.24	4	100
**GMO_EU_2008.tab**	557.78	376.73**	3	86	301.21	6	100
**GMO_EU_2010.tab**	1256.72	561.72**	3	96	429.06*	9*	100*
**GMO_EU_2012.tab**	1701.5	861.49**	3	91	592.84*	12*	99*
**GMO_all_2012.tab**	5280.92	No solution	No solution	No solution	1907.30*	14*	82*

*Interrupted before optimal result found.

**GMOtrack constrains parameters set to m = 3 (number of screening assays in solution) and coverage = 80% (the minimal coverage (in percentage of the total GM events in the dataset) of screening assay combinations).

Expected cost (event-specific assays only): cost (in arbitrary unit) of a sample analysis if no screening strategy is followed and only event-specific assays are use.

Lowest analytical cost GMOtrack: cost (in arbitrary unit) of a sample analysis using the best screening assay combination proposed by GMOtrack.

Combination GMOtrack (number assays): number of screening assays to be performed when following the best screening assay combination proposed by GMOtrack.

Coverage GMOtrack (%): Coverage (in percentage of the total GM events in the dataset) of the best screening assay combination proposed by GMOtrack.

Lowest analytical cost GMOseek: cost (in arbitrary unit) of a sample analysis using the best screening assay combination proposed by GMOseek.

Combination GMOseek (number assays): number of screening assays to be performed when following the best screening assay combination proposed by GMOseek.

Coverage GMOseek (%): Coverage (in percentage of the total GM events in the dataset) of the best screening assay combination proposed by GMOseek.

As for GMOtrack, the low frequency of a given GM event does not mean that its genetic elements (target for potential screening assays) are ignored in the algorithm. The algorithm is designed in such that the coverage factor parameter is on an equal footing with the cost parameter.

### Data acquisition and data subsets

The data regarding the known GM events relevant to the food, feed and seed legislation and their genetic components were collected during the European ERA-NET GMOseek project, and were recently made publicly available [8].

These data were then transferred into a tabulated matrix format, required by the GMOseek software (compatible with the GMOtrack format). Frequencies of presence of each GM event for the years 2006 to 2012 were gathered based on the actual observation in our routine GMO testing laboratory ("TestLab") and according to the following data and rules. Information about the GMO authorization status through the years was obtained from the GMO database hosted by the GMO Compass website ( http://www.gmo-compass.org/eng/gmo/db/) and from the European Food Safety Authority (EFSA) register of questions ( http://registerofquestions.efsa.europa.eu/roqFrontend/questionsListLoader?panel=GMO&questiontype=2). Information regarding the presence of unauthorized GMOs (UGMs) in the European Union (EU) was gathered from the Rapid Alert System for Food and Feed (RASFF) portal ( https://webgate.ec.europa.eu/rasff-window/portal/), and from the European Network of GMO Laboratories (ENGL).

If an UGM was never observed in EU, it was assigned a low probability of one per thousand (0.001). A frequency of 1% was attributed to UGMs already reported in the EU. All GM events authorized, tolerated [25], or under the so-called "low level presence for feed" regulation in the EU (EC 619/2011) [24], and thereafter termed EU GMO, observed with a frequency below 1% were given a 1% frequency in the dataset. All EU GM events observed with a frequency above 1% were attributed the actual frequency observed by the TestLab. All the collected datasets, assembled by years and by sample matrix type (food, feed, seed, all matrices), are available on the website ( http://www.gmoseek.com/gmoseek).

The GMOseek system is designed to detect all (known) GMOs. Frequency estimates are used (only) to compute the probabilities of outcomes of screening assays which are in turn used to estimate the total expected cost of a combination of screening assays. As it was the case for GMOtrack [22], the low frequency of a given GM event does not mean that its genetic elements (target for potential screening assays) are ignored in the algorithm. The algorithm is designed in a way that the coverage of all GM events is of utmost importance, while the cost estimate is used for strategy selection. The goal of this paper is to propose a very versatile tool and to exemplify its performance on real-world data. Therefore, users of GMOseek are recommended to use the data from their own testing history, or, alternatively, data coming from international studies.

In the following sections are described subsets of data employed to perform simulations runs with GMOseek. These simulations performed selecting all the species present in the tested datasets were intended to test the performance, the robustness and the relevance of the GMOseek package. Note that in the datasets, the software and this manuscript, the terms "corn" and "maize" are identical and refer to the same *Zea mays* species.

### Subsets for GMOtrack vs. GMOseek comparison

For comparing the GMOseek and GMOtrack software, the datasets GMO_EU_2005.tab and GMO_EU_2008.tab (Additional files 1 and 2) previously used for the validation of the GMOtrack software were utilized. Additional datasets with increasing data amount for years 2010 and 2012 and for different geographical zone (EU related GM events only, all GM events known globally) were prepared from the GMOseek project matrix [8] to be used with both the GMOseek and GMOtrack software (Additional files 3, 4 and 5). The data set size is indicated in Table 1. GMOtrack simulations were performed with the default settings (maximum five assays in the screening phase, minimum 80% coverage of all the GM events in the dataset in the screening phase). As GMOtrack becomes very limited with growing dataset, this 80% coverage parameter was chosen to speed-up the generation of results.

### Subsets for GMOseek robustness assessment

To assess whether the change in frequency of appearance of GM events would have an influence on the GMOseek algorithm robustness and the combinations of assays it proposes, several data subsets for EU GM events were prepared, then tested and compared with the subset containing the actual observed frequencies ("template data set", Additional file 6).

### Equal frequencies

In one experiment, all GM event frequencies were set at the same level (0.1%, 1%, 2%, 5% and 10%, respectively) (Additional files 7, 8, 9, 10 and 11). In another experiment ("Near future"), several scenarios for future situations were tested (near future 1, 2 and 3) and for each scenario, evolution of frequency (increase of GMO occurrence) was also tested.

#### Near future 1 subsets

These subsets (Additional files 12, 13, 14 and 15) were created to simulate a probable future situation in EU. In these subsets, the following modifications were made to the template EU GMO dataset (Additional file 6):

1. Increase the percentage of all EU authorized/in pipeline GM events from 1% (or their actual frequency) to 2%, 5% and 10%, respectively.
2. Decrease the frequency of UGMs found in EU to 0.1%.
3. Lower the frequency of the tolerated GM events to 0.1%.
4. The actual GTS 40-3-2, most widely planted GMO in the world (RoundUp Ready soybean – termed "RRS", in the following) frequency (46%) stays the same.

#### Near future 2 subsets

These subsets are similar as the above described ones (near future 1). In these subsets (Additional files 16, 17, 18 and 19), the EU GM events for which the first application for authorization was submitted in 2003 or before (considered as "old GM events") see their frequency stagnating. RRS frequency decreases to 25%, and only the newer events (first application in EU after 2003) see their frequency increasing from 1% to 2%, 5% and 10%. Data regarding the authorization status and submission dates were obtained from the Community register of genetically modified food and feed ( http://ec.europa.eu/food/dyna/gm_register/index_en.cfm) and from the GMO compass database ( http://www.gmo-compass.org/eng/gmo/db/).

#### Near future 3 subsets

These are the same subsets as for near future 2 with the "old" events (first application for authorization in EU from 2003 or before) having their frequency decreased to 0.5%, and RRS frequency decreased to 5%. In these subsets (Additional files 20, 21, 22 and 23), only the frequencies for the new events (authorization dossier in EU submitted after 2003) increase from 1% to 2%, 5% and 10%. Two additional files (Additional files 23bis and 23ter) were created based on the last mentioned dataset (new events at 10%) with one GM event frequency being set at 80%, and another dataset with four GM event found with 80% frequency.

### 5plex subset

In this subset of a template dataset (Additional file 24), only the genetic components proposed by Waiblinger and collaborators [12] in their practical approach for detecting GMOs are targeted, especially because also the pentaplex (5plex) method was recently published [26]. This 5plex had a considerable impact on the implementation of the matrix based approach within the community of GMO testing laboratories. This subset (Additional file 25) was created and used to compare the performance of the 5plex screening strategy with the screening strategies proposed by the GMOseek algorithm.

## GMOseek test results

### Robustness to frequency changes

To assess whether the change in frequency of appearance of GMO events would have an influence on the GMOseek algorithm robustness and the combinations of assays it proposes, several data subsets for GMOs authorized, tolerated or in pipeline in EU were tested. In one experiment ("equal frequencies"), all GM events were set at the same frequencies ranging from 0.1% to 10%. In another experiment ("Near future"), several scenarios for future situations were tested (near future 1, 2 and 3) and for each scenario, the evolution of frequency (increase of GMO occurrence) was also tested.

#### Equal frequencies

Details on the results of computation can be found in Additional file 26. For the currently EU authorized, tolerated and in pipeline GMOs, the change of frequencies has low influence on the GMO coverage and the proposed combinations of screening assays (frequency at 1% and 2%). The main observed change is the expected cost saving for the best combination (the lower is the frequency, the larger is the cost saving).

With very low presence of all GM events (0.1%), the screening combination would be simpler with four screening elements to be targeted instead of nine genetic components for frequencies at 1% and 2%). At higher frequencies (5% and 10%), larger combinations of screening assays (similar to the previous ones but with additional assays) are needed and the expected saving on analysis cost rapidly decreases. However, the GMO coverage is maintained at the same level.

The GMOseek algorithm is able to handle relatively high percentage of GMO frequencies. The effect of frequency change on GMO coverage is moderate but the higher is the frequency, the lower is the cost saving provided by the screening combinations. However, it must be observed that even when EU authorized and in pipeline GM events are very frequent (10%), very good coverage (98%) and significant savings (close to 30% of the initial costs) can be obtained using GMOseek. Note that in some cases (current GMO frequencies, 5%, 10%), the best combination proposed by GMOseek provides only little advantage in terms of cost savings compared to the previously proposed assay combination(s) for similar GMO coverage.

#### Near Future 1

In this scenario, at the first frequency level (1%), the coverage and expected cost saving of the best combinations are comparable with those observed with combination obtained using the "template data set". Logically, the expected cost saving decreases rapidly with increasing GMO frequency while coverage and proposed combinations remains mostly unchanged. However, it must be observed that even when EU authorized and in-pipeline GMOs are very frequent (10%), very good coverage and significant savings can be obtained using GMOseek. Further details on the results for these simulations can be found in Additional file 27.

#### Near Future 2

In this scenario, at the first frequency level (1%), the coverage and expected cost saving of the best combinations are better than those observed for the combination obtained with the "template data set" and for the near future 1 scenario. Also for the other level of GMO frequency, for similar coverage and combinations, better cost savings are expected than for the near future 1 scenario. Logically, the expected cost saving decreases rapidly with increasing GMO frequency while coverage and proposed combinations remains mostly unchanged. However, it must be observed that even when EU authorized and in-pipeline GMOs are very frequent (10%), very good coverage and significant savings can be obtained using GMOseek. Further details on the results for these simulations can be found in Additional file 28.

#### Near Future 3

Results for this scenario in terms of proposed combination, cost saving and GMO coverage are quite comparable to those of the scenario near future 2. Further details on the results for these simulations can be found in Additional file 29.

As a conclusion of the simulations done based in these scenarios, using GMOseek for choosing the best combinations of screening assays (and therefore developing new assays for future GMO status) always shows good results in terms of GMO coverage and cost saving. These simulations demonstrate the robustness of the algorithm as well as the validity of using carefully chosen combinations of screening assays for accurate and cost-efficient GMO detection. Moreover, even with a very high frequency of GM events (80%) as it is sometimes observed in feed samples, GMOseek is still able to propose combinations enabling significant cost savings. These results demonstrate that the GMOseek algorithm could be a long lasting tool for helping the analyst involved in GMO diagnostics.

### Relevance of the results: test on real samples

**Figure 4:**
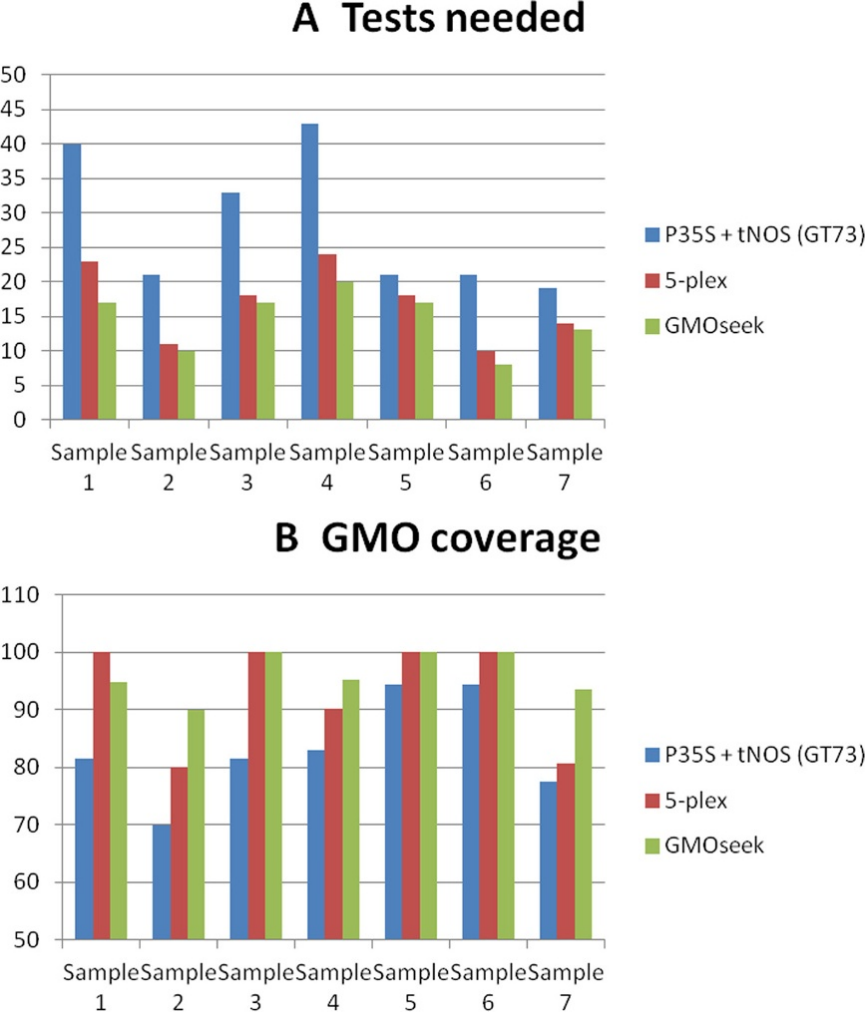
**Comparison of the GMOseek, 5plex and conventional screening strategies performance. A**: Total number of tests needed to identify the GM events in the samples. Vertical axis: number of tests used during the screening and identification phases. **B**: Coverage of the tested screening strategies. Vertical axis: % of the GM events of the dataset that are covered by the screening phase.

Some real routine samples analyzed by the TestLab were selected to evaluate the relevance of the matrix approach proposed by GMOseek. To do so, the cost (directly linked to the number of tests needed) and coverage of the conventional testing strategy (screening the P35S and T-*nos* and optionally GT73 genetic components, followed by event-specific analysis) previously used by the TestLab were compared with those of the strategy proposed by GMOseek. Comparison was also made with the previously described fixed five-components screening strategy (5plex) [12] making use of the 5plex data subsets. Methods and results of these simulations are available in Additional file 30.The optimal combination proposed by GMOseek always offers better coverage (with one exception) and better cost-efficiency than the 5plex combination approach. In all cases, both GMOseek and 5plex combinations provide better coverage and cost-efficiency than the P35S x t-NOS (and sometimes GT73) screening phase previously used by the TestLab (Figure 4). Therefore, the use of GMOseek is relevant when tested on real samples and would, in every case, allow better cost efficiency for an equal or better GMO coverage would it be used for routine GMO testing thanks to its superior screening strategy and the DSS leading to an easy interpretation of the testing results. Moreover, the algorithm would be able to warn about the discrepancies between the screening phase and identification results, observed in two samples.

In routine analysis, screening a set of samples is most commonly practiced. In practice, test labs usually receive a batch of samples of the same type, or alternatively, if different test samples are submitted, they often have the same ingredients. In these cases, a single strategy should be on all samples. If, in contrary, samples to be tested are diverse, GMOseek proposes several combinations of assays with near-optimal cost and coverage for each sample. It is very straight forward to look for common combination of assays satisfying the coverage and cost targets set by the test laboratory. Moreover, one can even adapt the combination proposed by right-clicking the assays in the inspection panel. In conclusion and based on the authors experience with the tool, the software is well tuned to routine analyses.

### Performance of GMOseek compared to GMOtrack

In all cases, GMOseek provides results leading to cheaper total analytical costs with at least equal coverage of the GM events. The larger the dataset, the superior are the cost-efficiency and the coverage of the screening combinations offered by GMOseek in comparison with those proposed by GMOtrack. This observation is not a surprise as the search strategy of GMOtrack restricts the number of combinations to evaluate and therefore the number of assays to be performed in the screening phase. For this reason, the default maximum number of assays is set to five in GMOtrack. The constraint optimization-based search strategy adopted for GMOseek allows to submit much larger datasets, scrutinizing larger combinations and to propose larger sets of screening assays. Consequently, cheaper solution using more informative, larger sets of assays covering more GM events can be obtained in a practical timeframe (from a few seconds to a few minutes). As such, in addition to the availability of a user-friendly graphical interface and a decision support system, GMOseek proves to be the right tool to suggest combinations of screening assays.

## Conclusions

The GMOseek software is a multifunction tool, proven to facilitate routine analysis of GMOs in food, feed or seed samples. GMOseek also provides a user-friendly interface with a decision support system which guides the user through all three steps of the cost-efficient matrix GMO testing approach: from the selection of screening assays, deciding on the event-specific assays to be performed and the final interpretation of results. Due to its ability to simulate testing costs in future scenarios, it can be used not only for routine laboratory testing, but also for research. It is adapted to the current situation of GMOs commercialized worldwide and the algorithm is robust to face the future changes in the status of GMOs. It is easily tuned to new situations on the market by changing the input data matrix. Thanks to its flexibility and user-friendly interface, it should easily find its way in GMO testing and methods developing laboratories.

Possible improvements of the GMOseek system would be a direct connection with the EUginius molecular registry ( http://euginius.eu/) currently in development, which should keep updated the knowledge of the GM events commercialized or in pipeline, worldwide, as well as their genetic components. With such link to the molecular registry, automatic skimming of the genetic components originating from the host species could be set-up to optimize the combinations of screening assays. Finally, a new functionality taking into account the increasing need to perform multiplex tests (targeting several DNA sequences simultaneously) should be incorporated in the software to refine the cost-efficiency calculation.

### Availability and requirements

The GMOseek software is packed and deployed as one file which is named GMOseek.jar. It can be placed at any location suitable for a user. The program can be executed by a double click on the file from a file manager or by java -jar GMOseek.jar from the command line. The software runs on any operating system which has java 1.5 or later installed. The software is freely available at http://www.gmoseek.com/gmoseek. The documentation files to facilitate GMOseek use are available at the same URL.

**Project name**: GMOseek

**Project home pages**: http://www.gmoseek.com/gmoseek

**Operating system(s):** Platform independent which has java 1.5 or later installed.

**Programming language:** Java

**Other requirements:** No.

**License:** GPL

**Any restrictions to use:** GMOseek is open source software issued under the GNU General Public License.

## Electronic supplementary material

Additional file 1: **GMO_EU_2005.tab.** Tabulated file with frequencies and genetic components of the different EU authorized GM events in 2005. (ZIP 3 KB)

Additional file 2: **GMO_EU_2008.tab.** Tabulated file with frequencies and genetic components of the different EU authorized GM events in 2008. (ZIP 10 KB)

Additional file 3: **GMO_EU_2010.tab.** Tabulated file with frequencies and genetic components of the different EU authorized GM events in 2010. (ZIP 2 KB)

Additional file 4: **GMO_EU_2012.tab.** Tabulated file with frequencies and genetic components of the different EU authorized GM events in 2012. (ZIP 2 KB)

Additional file 5: **GMO_all_2012.tab.** Tabulated file with frequencies and genetic components of all known GM events in 2012. (ZIP 2 KB)

Additional file 6: **Actual frequency EU.tab.** Tabulated file with TestLab observed frequencies used for robustness test. (ZIP 2 KB)

Additional file 7: **EUv0_1.tab.** Tabulated file with all GMO frequencies from the actual frequency EU.tab file set to 0.1%. Used for robustness test. (ZIP 2 KB)

Additional file 8: **EUv1.tab.** Tabulated file with all GMO frequencies from the actual frequency EU.tab file set to 1%. Used for robustness test. (ZIP 2 KB)

Additional file 9: **EUv2.tab.** Tabulated file with all GMO frequencies from the actual frequency EU.tab file set to 2%. Used for robustness test. (ZIP 1 KB)

Additional file 10: **EUv5.tab.** Tabulated file with all GMO frequencies from the actual frequency EU.tab file set to 5%. Used for robustness test. (ZIP 1 KB)

Additional file 11: **EUv10.tab.** Tabulated file with all GMO frequencies from the actual frequency EU.tab file set to 10%. Used for robustness test. (ZIP 1 KB)

Additional file 12: **EUnearfuture1v1.tab.** Tabulated file simulating near future scenario 1 with EU GMO frequencies set to 1%. Used for robustness test. (ZIP 1 KB)

Additional file 13: **EUnearfuture1v2.tab.** Tabulated file simulating near future scenario 1 with EU GMO frequencies set to 2%. Used for robustness test. (ZIP 2 KB)

Additional file 14: **EUnearfuture1v5.tab.** Tabulated file simulating near future scenario 1 with EU GMO frequencies set to 5%. Used for robustness test. (ZIP 2 KB)

Additional file 15: **EUnearfuture1v10.tab.** Tabulated file simulating near future scenario 1 with EU GMO frequencies set to 10%. Used for robustness test. (ZIP 2 KB)

Additional file 16: **EUnearfuture2v1.tab.** Tabulated file simulating near future scenario 2 with EU GMO frequencies set to 1%. Used for robustness test. (ZIP 2 KB)

Additional file 17: **EUnearfuture2v2.tab.** Tabulated file simulating near future scenario 2 with EU GMO frequencies set to 2%. Used for robustness test. (ZIP 1 KB)

Additional file 18: **EUnearfuture2v5.tab.** Tabulated file simulating near future scenario 2 with EU GMO frequencies set to 5%. Used for robustness test. (ZIP 2 KB)

Additional file 19: **EUnearfuture2v10.tab.** Tabulated file simulating near future scenario 2 with EU GMO frequencies set to 10%. Used for robustness test. (ZIP 1 KB)

Additional file 20: **EUnearfuture3v1.tab.** Tabulated file simulating near future scenario 3 with EU GMO frequencies set to 1%. Used for robustness test. (ZIP 1 KB)

Additional file 21: **EUnearfuture3v2.tab.** Tabulated file simulating near future scenario3 with EU GMO frequencies set to 2%. Used for robustness test. (ZIP 2 KB)

Additional file 22: **EUnearfuture3v5.tab.** Tabulated file simulating near future scenario 3 with EU GMO frequencies set to 5%. Used for robustness test. (ZIP 1 KB)

Additional file 23: **EUnearfuture3v10.tab.** Tabulated file simulating near future scenario 3 with EU GMO frequencies set to 10%. Used for robustness test. .bis: EUnearfuture3v80_1.tab. Tabulated file simulating near future scenario 3 with EU GMO frequencies set to 10% and one GM event at 80%. Used for robustness test. ter: EUnearfuture3v80_4.tab. Tabulated file simulating near future scenario 3 with EU GMO frequencies set to 10% and four GM events at 80%. Used for robustness test. (ZIP 1 KB)

Additional file 24: **Template_5plex_test.tab.** Tabulated file used as template to create the 5plex subset. Used for comparing the GMOseek, 5plex and old screening strategies. (PDF 399 KB)

Additional file 25: **Dataset_5_components.tab.** Tabulated file used to perform the 5plex strategy evaluation. Used for comparing the GMOseek, 5plex and old screening strategies. (PDF 785 KB)

Additional file 26: **Raw results of the GMOseek robustness tests performed with equal frequencies.** (PDF 219 KB)

Additional file 27: **Raw results of the GMOseek robustness tests performed with the scenario "near future 1".** (PDF 399 KB)

Additional file 28: **Raw results of the GMOseek robustness tests performed with the scenario "near future 2".** (PDF 785 KB)

Additional file 29: **Raw results of the GMOseek robustness tests performed with the scenario "near future 3".** (ZIP 1 KB)

Additional file 30: **Methods and results of the comparison between different screening strategies: previous P35S + t-NOS (GT73), 5plex approach, GMOseek.** (ZIP 1 KB)
