# Supplementary material for: GMOseek: a user friendly tool for optimized GMO testing
Source: BMC Bioinformatics. 2014 Aug 1;15(1):258. doi: 10.1186/1471-2105-15-258 (PMC4138379; doi:10.1186/1471-2105-15-258)
Supplement: Supplementary file 24 — Additional file 24: Template_5plex_test.tab. Tabulated file used as template to create the 5plex subset. Used for comparing the GMOseek, 5plex and old screening strategies. (PDF 399 KB) [file 12859_2013_6540_MOESM24_ESM.pdf]

Explanation

Below are given the combinations proposed by the GMOseek algorithm after simulations on subsets of the template EU GMO matrix. Description regarding these subsets is given in the section Near future 3 subsets of the deliverable D3/01.

Each simulation was interrupted after a long time of computation, meaning that the optimal solution is probably the last one found by the algorithm.

Each solution is provided as follows:

Time for completion (in milliseconds, ms)

Expected cost of the whole analysis (taking in account the screening phase and the identification phase costs)

(Expected cost of the whole analysis if only event-specific methods, cost saving in %)

Proposed combination (genetic elements to be targeted) (coverage of the solution: % coverage, ratio covered GM events/GM events in the matrix)

In the example below, the combination was provided in 203ms, expected cost is 1120,95 units while the cost using only event-specific method would be 1256.72. Therefore, expected savings on cost of analysis is 10.8%. The solution is a screening phase with P-35S only that covers 55% of the GM events to be analysed i.e. 30 of the 55 GM events in the matrix. The computation was interrupted after 37005000ms.

Example:

203ms Solution: 1120.95 (1256.72, 10.8%)

[P-35s] (55%, 30/55)

3705000ms

Interrupted!

Note that for the two last frequency levels, the best combination provide only low gain in terms of cost savings compared to the previously proposed combination(s) for the same GMO coverage.

First frequency level (1%)

156ms Solution: 716.83 (1023.74, 29.98%)

[P-35s] (57%, 25/44)

313ms Solution: 577.9 (1023.74, 43.55%)

[P-35s] [T-nos] (75%, 33/44)

563ms Solution: 489.87 (1023.74, 52.15%)

[P-35s] [T-E9] [T-nos] (86%, 38/44)

906ms Solution: 446.01 (1023.74, 56.43%)

[P-35s] [T-E9] [T-nos] [pat] (91%, 40/44)

2094ms Solution: 443.12 (1023.74, 56.72%)

[P-35s] [P-Kti3] [P-ubiZM1] [T-E9] [T-nos] [m epsps] [pat] [CTP1] (98%, 43/44)

Covers all except BPS-CV127-9.

1649166ms

Interrupted!

Second frequency level (2%)

219ms Solution: 764.9 (1023.74, 25.28%)

[P-35s] (57%, 25/44)

750ms Solution: 629.04 (1023.74, 38.56%)

[P-35s] [T-nos] (75%, 33/44)

2079ms Solution: 542.06 (1023.74, 47.05%)

[P-35s] [T-E9] [T-nos] (86%, 38/44)

4016ms Solution: 491.51 (1023.74, 51.99%)

[P-35s] [T-E9] [T-nos] [pat] (91%, 40/44)

6172ms Solution: 490.82 (1023.74, 52.06%)

[P-35s] [P-ubiZM1] [T-E9] [T-nos] [pat] (91%, 40/44)

8876ms Solution: 488.33 (1023.74, 52.3%)

[P-35s] [P-ubiZM1] [T-E9] [T-nos] [pat] [CTP1] (93%, 41/44)

12235ms Solution: 483.34 (1023.74, 52.79%)

[P-35s] [P-Kti3] [P-ubiZM1] [T-E9] [T-nos] [pat] [CTP1] (95%, 42/44)

16407ms Solution: 479.26 (1023.74, 53.19%)

[P-35s] [P-Kti3] [P-ubiZM1] [T-E9] [T-nos] [m epsps] [pat] [CTP1] (98%, 43/44)

Covers all except BPS-CV127-9.

1743864ms

Interrupted!

Third frequency level (5%)

313ms Solution: 880.36 (1023.74, 14.01%)

[P-35s] (57%, 25/44)

1563ms Solution: 762.35 (1023.74, 25.53%)

[P-35s] [T-nos] (75%, 33/44)

5391ms Solution: 680.56 (1023.74, 33.52%)

[P-35s] [T-E9] [T-nos] (86%, 38/44)

11500ms Solution: 618.64 (1023.74, 39.57%)

[P-35s] [T-E9] [T-nos] [pat] (91%, 40/44)

18625ms Solution: 607.76 (1023.74, 40.63%)

[P-35s] [P-ubiZM1] [T-E9] [T-nos] [pat] (91%, 40/44)

27891ms Solution: 600.08 (1023.74, 41.38%)

[P-35s] [P-ubiZM1] [T-E9] [T-nos] [pat] [CTP1] (93%, 41/44)

39969ms Solution: 592.36 (1023.74, 42.14%)

[P-35s] [P-Kti3] [P-ubiZM1] [T-E9] [T-nos] [pat] [CTP1] (95%, 42/44)

55595ms Solution: 585.91 (1023.74, 42.77%)

[P-35s] [P-Kti3] [P-ubiZM1] [T-E9] [T-nos] [m epsps] [pat] [CTP1] (98%, 43/44)

72142ms Solution: 585.46 (1023.74, 42.81%)

[P-35s] [P-Kti3] [P-ubiZM1] [T-E9] [T-nos] [m epsps] [pat] [CTP1] [CTP2-CP4EPSPS] (98%, 43/44)

89626ms Solution: 582.85 (1023.74, 43.07%)

[P-35s] [P-Kti3] [P-ubiZM1] [T-E9] [T-nos] [m epsps] [pat] [bar] [CTP1] [CTP2-CP4EPSPS] (98%, 43/44)

108392ms Solution: 575.81 (1023.74, 43.75%)

[P-35s] [P-Kti3] [P-ubiZM1] [T-E9] [T-nos] [CryIaB / CryIac] [m epsps] [pat] [bar] [CTP1] [CTP2-CP4EPSPS] (98%, 43/44)

Covers all except BPS-CV127-9

1546797ms

Interrupted!

Fourth frequency level (10%)

610ms Solution: 1002.41 (1023.74, 2.08%)

[P-35s] (57%, 25/44)

5375ms Solution: 923.81 (1023.74, 9.76%)

[P-35s] [T-nos] (75%, 33/44)

24657ms Solution: 854.96 (1023.74, 16.49%)

[P-35s] [T-E9] [T-nos] (86%, 38/44)

60628ms Solution: 793.03 (1023.74, 22.54%)

[P-35s] [T-E9] [T-nos] [pat] (91%, 40/44)

103536ms Solution: 778.92 (1023.74, 23.91%)

[P-35s] [P-ubiZM1] [T-E9] [T-nos] [pat] (91%, 40/44)

166491ms Solution: 762.08 (1023.74, 25.56%)

[P-35s] [P-ubiZM1] [T-E9] [T-nos] [pat] [CTP1] (93%, 41/44)

256165ms Solution: 752.42 (1023.74, 26.5%)

[P-35s] [P-Kti3] [P-ubiZM1] [T-E9] [T-nos] [pat] [CTP1] (95%, 42/44)

380606ms Solution: 743.93 (1023.74, 27.33%)

[P-35s] [P-Kti3] [P-ubiZM1] [T-E9] [T-nos] [m epsps] [pat] [CTP1] (98%, 43/44)

514280ms Solution: 734.55 (1023.74, 28.25%)

[P-35s] [P-Kti3] [P-ubiZM1] [T-E9] [T-nos] [m epsps] [pat] [CTP1] [CTP2-CP4EPSPS] (98%, 43/44)

657922ms Solution: 721.03 (1023.74, 29.57%)

[P-35s] [P-Kti3] [P-ubiZM1] [T-E9] [T-nos] [m epsps] [pat] [bar] [CTP1] [CTP2-CP4EPSPS] (98%, 43/44)

812720ms Solution: 696.72 (1023.74, 31.94%)

[P-35s] [P-Kti3] [P-ubiZM1] [T-E9] [T-nos] [CryIaB / CryIac] [m epsps] [pat] [bar] [CTP1] [CTP2-CP4EPSPS] (98%, 43/44)

979971ms Solution: 687.19 (1023.74, 32.87%)

[P-35s] [P-Kti3] [P-ubiZM1] [T-E9] [T-nos] [CryIaB / CryIac] [m epsps] [nptII] [pat] [bar] [CTP1] [CTP2-CP4EPSPS] (98%, 43/44)

Covers all except BPS-CV127-9

3698397ms

Interrupted!

Fifth frequency = Fourth frequency level (10%) + one GMO at 80%

200ms

Solution: 1002.41 (1023.74, 2.08%)

[P-35s] (57%, 25/44)

1570ms

Solution: 923.81 (1023.74, 9.76%)

[P-35s] [T-nos] (75%, 33/44)

7396ms

Solution: 854.96 (1023.74, 16.49%)

[P-35s] [T-E9] [T-nos] (86%, 38/44)

18913ms

Solution: 793.03 (1023.74, 22.54%)

[P-35s] [T-E9] [T-nos] [pat] (91%, 40/44)

32175ms

Solution: 778.92 (1023.74, 23.91%)

[P-35s] [P-ubiZM1] [T-E9] [T-nos] [pat] (91%, 40/44)

93244ms

Solution: 777.66 (1023.74, 24.04%)

[P-35s] [P-Kti3] [P-ubiZM1] [T-E9] [T-nos] [pat] [CTP1] (95%, 42/44)

148175ms

Solution: 768.33 (1023.74, 24.95%)

[P-35s] [P-Kti3] [P-ubiZM1] [T-E9] [T-nos] [m epsps] [pat] [CTP1] (98%, 43/44)

208062ms

Solution: 756.29 (1023.74, 26.13%)

[P-35s] [P-Kti3] [P-ubiZM1] [T-E9] [T-nos] [m epsps] [pat] [CTP1] [CTP2-CP4EPSPS] (98%, 43/44)

277175ms

Solution: 742.92 (1023.74, 27.43%)

[P-35s] [P-Kti3] [P-ubiZM1] [T-E9] [T-nos] [m epsps] [pat] [bar] [CTP1] [CTP2-CP4EPSPS] (98%, 43/44)

354159ms

Solution: 718.86 (1023.74, 29.78%)

[P-35s] [P-Kti3] [P-ubiZM1] [T-E9] [T-nos] [CryIaB / CryIac] [m epsps] [pat] [bar] [CTP1] [CTP2-CP4EPSPS] (98%, 43/44)

437298ms

Solution: 707.91 (1023.74, 30.85%)

[P-35s] [P-Kti3] [P-ubiZM1] [T-E9] [T-nos] [CryIaB / CryIac] [m epsps] [nptII] [pat] [bar] [CTP1] [CTP2-CP4EPSPS] (98%, 43/44)

Covers all except BPS-CV127-9

522989ms

Interrupted!

Sixth frequency = Fourth frequency level (10%) + four GMOs at 80%

190ms

Solution: 1002.41 (1023.74, 2.08%)

[P-35s] (57%, 25/44)

1552ms

Solution: 923.81 (1023.74, 9.76%)

[P-35s] [T-nos] (75%, 33/44)

41064ms

Solution: 879.43 (1023.74, 14.1%)

[P-35s] [T-E9] [T-nos] [pat] (91%, 40/44)

73026ms

Solution: 865.25 (1023.74, 15.48%)

[P-35s] [P-ubiZM1] [T-E9] [T-nos] [pat] (91%, 40/44)

245989ms

Solution: 862.86 (1023.74, 15.71%)

[P-35s] [P-Kti3] [P-ubiZM1] [T-E9] [T-nos] [pat] [CTP1] (95%, 42/44)

402211ms

Solution: 853.97 (1023.74, 16.58%)

[P-35s] [P-Kti3] [P-ubiZM1] [T-E9] [T-nos] [m epsps] [pat] [CTP1] (98%, 43/44)

754992ms

Solution: 851.81 (1023.74, 16.79%)

[P-35s] [P-Kti3] [P-ubiZM1] [T-E9] [T-nos] [m epsps] [pat] [bar] [CTP1] [CTP2-CP4EPSPS] (98%, 43/44)

974375ms

Solution: 827.29 (1023.74, 19.19%)

[P-35s] [P-Kti3] [P-ubiZM1] [T-E9] [T-nos] [CryIaB / CryIac] [m epsps] [pat] [bar] [CTP1] [CTP2-CP4EPSPS] (98%, 43/44)

1217712ms

Solution: 811.68 (1023.74, 20.71%)

[P-35s] [P-Kti3] [P-ubiZM1] [T-E9] [T-nos] [CryIaB / CryIac] [m epsps] [nptII] [pat] [bar] [CTP1] [CTP2-CP4EPSPS] (98%, 43/44)

Covers all except BPS-CV127-9

1463036ms

Interrupted!
