# Supplementary material for: GMOseek: a user friendly tool for optimized GMO testing
Source: BMC Bioinformatics. 2014 Aug 1;15(1):258. doi: 10.1186/1471-2105-15-258 (PMC4138379; doi:10.1186/1471-2105-15-258)
Supplement: Supplementary file 25 — Additional file 25: Dataset_5_components.tab. Tabulated file used to perform the 5plex strategy evaluation. Used for comparing the GMOseek, 5plex and old screening strategies. (PDF 785 KB) [file 12859_2013_6540_MOESM25_ESM.pdf]

Explanation

Current screening approach: previously, the TestLab (Slovenian national reference laboratory for GMO diagnostics hosted in NIB) was following a simple [P-35S] x [T-nos] screening approach (with an additional [GT73] event-specific test when oilseed rape was in the sample). However, this screening only covered a part of the EU authorized, tolerated or in pipeline GMO events.

Sample analysis and matrix subset: the customers or inspection services target the analysis made by the test laboratory. They usually ask to check for the presence of the EU authorized, tolerated or in pipeline GM events only (as well as a limited number of UGM lines already found in EU). Therefore, the simulations were done based on subsets of the matrix containing EU authorized, tolerated or in pipeline GM events as well as these UGM events.

Note: when the simulations were done, the maize event LY038 was in pipeline for authorization. This event was withdrawn from application list since then.

Results: As the samples described in this appendix were already analysed, we decided to take as cost reference the number of assays that would have been needed if the screening combinations proposed by GMOseek algorithm or the 5-plex approach would have been used. This number of assays is compared to the number of assays that was actually used by the test laboratory.

In the last page of this file is given the result of a cost comparison of the two “same strategy for all samples” (current approach at TestLab and 5plex approach) and the “sample-centered GMO testing strategy” (GMOseek combinations) for the seven samples analyzed below.

Sample 1

Background:

In the test sample 1, it was asked to use the triple screening T-nos, P35S, RT 73, followed by identification of all possible authorized, tolerated or in pipeline maize, soybean and oilseed rape lines in EU (including the UGM event 32 and Bt10). This resulted in 3 screening tests + 37 event-specific tests (excluding RT73 tested in screening phase). The final conclusion was presence of RRS only. **So, total of 40 qualitative tests. The screening approach covers 31 of the 38 possible GMOs.**

Optimal combination proposed by GMOseek:

[P-35s] [P-ract] [T-35s] [T-E9] [T-nos] [pat] [bar] [CTP1]. Combination covers 36 out of 38 GMOs. Not covered: 305423, BPS-CV127-9.

Theoretically saves 43% of the costs of analysis.

As only P-35S and T-nos are positive in this screening, we need to check for the presence of 89034, 98140, MIR604, Mon810, Mon863, 305423, 356043, BPS-CV127-9 and RRS. Only RRS is positive.

Conclusion: 8 screening tests plus 9 event-specific tests = **total of 17 qualitative tests** (**57.5% saving in terms of tests**)

Alternative combination:

A good alternative to the optimal solution is the following one, also proposed by GMOseek **[P-35s] [P-ract] [T-E9] [T-nos] [pat] [bar] [CTP1]. Combination covers 36 out of 38 GMOs. Not covered: 305423, BPS-CV127-9.** In comparison to the optimal solution, it allows the same coverage with 42.2% of expected savings in cost analysis (when comparing to 43% for the optimal solution).

As only P-35S and T-nos are positive in this screening, we need to check for the presence of 89034, 98140, MIR604, Mon810, Mon863, 305423, 356043, BPS-CV127-9 and RRS. Only RRS is positive.

Conclusion: 7 screening tests plus 9 event-specific tests = **total of 16 qualitative tests** (**60% saving in terms of tests**)

5plex combination:

[T-nos] [P35S] [P35S-pat] [CTP2-CP4EPSPS] [bar]. Combination covers all GMOs.

As only P-35S and T-nos are positive in this screening, we need to check for the presence of 87460, 89034, Bt10 (UGM), event 32 (UGM), event 3272, 98140, GA21, LY038, Mir162, MIR604, Mon810, Mon863, 305423, 356043, BPS-CV127-9, RRS, Mon87701 and Mon87769. Only RRS is positive.

Conclusion: 5 screening tests plus 18 event-specific tests = **total of 23 qualitative tests** (**42.5% saving in terms of tests**).

Both combinations from GMOseek are informative and cost efficient and provide better coverage and cost-efficiency than the 5plex approach. All combinations provide substantial saving in terms of assays needed, when comparing to the current approach.

Sample 2

Background:

In the test sample 2, it was asked to use the triple screening T-nos, P35S, RT 73, followed by identification of all possible authorized, tolerated and in pipeline oilseed rape and soybean lines in EU. This resulted in 3 screening tests + 18 event-specific tests (excluding RT73 tested in screening phase). The final conclusion was presence of RRS only. **So, total of 21 qualitative tests. 14 of the 20 possible GMO events are covered by the screening approach.**

Best combination proposed by GMOseek:

[P-35s] [P-SsuAra] [T-E9] [T-nos] [pat] [CTP1] Combination covers all GMOs but the events 305423 and BPS-CV127-9.

Theoretically saves 21.5% of the costs of analysis.

As only T-nos is positive in this screening, we need to check for the presence of 305423, 356043, BPS-CV127-9 and RRS. Only RRS is positive.

Conclusion: 6 screening tests plus 4 event-specific tests = **total of 10 qualitative tests** (**+52% saving in terms of tests**)

5plex combination:

[T-nos] [P35S] [P35S-pat] [CTP2-CP4EPSPS] [bar]. Combination covers all GMOs but 305423, BPS-CV127-9, Mon87701 and Mon87769.

As only T-nos and P-35S are positive in this screening, we need to check for the presence of 305423, 356043, BPS-CV127-9, RRS, Mon87701 and Mon87769. Only RRS is positive.

Conclusion: 5 screening tests plus 6 event-specific test = **total of 11 qualitative tests** (**48% saving in terms of tests**).

The combination from GMOseek is more informative and provides better coverage and cost-efficiency than the 5plex approach. Both combinations allow substantial savings when comparing to the current screening approach

Sample 3

Background:

In the test sample 3, it was asked to use the triple screening T-nos, P35S, RT 73, followed by identification of all possible authorized, tolerated and in pipeline maize, oilseed rape and sugar-beet lines in EU (including the UGM event 32 and Bt10). This resulted in 3 screening tests + 30 event-specific tests (excluding RT73 tested in screening phase). The final conclusion was no presence of tested GMOs. **So, total of 33 qualitative tests. In the end non-explainable P-35S and T-nos signals were observed. They were finally attributed to traces of RRS (soybean, not tested in the first phases) in the sample. The screening combination covers 31 of the 38 possible GMO events.**

Optimal combination proposed by GMOseek:

[P-35s] [P-ract] [T-nos] [pat] [CTP2-CP4EPSPS]. Combination covers all 38 GMOs.
Theoretically saves 61% of costs of analysis.
As T-nos and P35S are positive in this screening, we need to check for the presence of Rf1, Rf2, Rf3, 89034, 176, event 3272, event 98140, MIR162, MIR604, Mon810, Mon863, and A5-15. All negative. ***Note: the GMOseek algorithm then alerts that the results of screening phase are in contradiction with those of the identification phase.***
Conclusion: 5 screening tests plus 12 event-specific tests = **total of 17 qualitative tests** (**48% saving in terms of tests**)

5plex combination:

[T-nos] [P35S] [P35S-pat] [CTP2-CP4EPSPS] [bar]. Combination covers all GMOs.
As only T-nos and P-35S are positive in this screening, we need to check for the presence of 87460, 89034, Bt10 (UGM), event 32 (UGM), event 3272, event 98140, GA21, LY038, MIR162, MIR604, Mon810, Mon863, and A5-15. All negative. *Note: the GMOseek algorithm then alerts that the results of screening phase are in contradiction with those of the identification phase.*
Conclusion: 5 screening tests plus 13 event-specific tests = **total of 18 qualitative tests** (**45.5% saving in terms of tests**).

The combination from GMOseek is more informative and provides better coverage and cost-efficiency than the 5plex combination. Both combinations allow substantial savings when comparing to the current screening approach

Sample 4

Background:

In the test sample 4, it was asked to use the triple screening T-nos, P35S, RT 73, followed by identification of all possible authorized, tolerated and in pipeline maize, soybean, oilseed rape and rice lines (including the UGM LL601, Bt10, event 32 and Bt63) in EU. This resulted in 3 screening tests + 40 event-specific tests (excluding RT73 tested in screening phase). The final conclusion was presence of RRS only. **So, total of 43 qualitative tests. The screening approach covers 34 of the 41 possible GMOs in the EU GMO dataset.**

Optimal combination proposed by GMOseek:

[P-35s] [T-35s] [T-E9] [T-nos] [T-pinII] [cry1Ac] [pat] [bar] [CTP2-CP4EPSPS]. Combination covers all GMOs but 305423 and BPS-CV127-9.

Theoretically saves 43% of costs of analysis

As only P-35S and T-nos are positive in this screening, we need to check for the presence of 87460, 89034, GA21, LY038, MIR162, MIR604, Mon810, Mon863, 305423, RRS and BPS-CV127-9. Only RRS is positive.

Conclusion: 9 screening tests plus 11 event-specific tests = **total of 20 qualitative tests** (**53.5% saving in terms of tests**)

Alternative combination:

A good alternative to the optimal solution is the following one, also proposed by GMOseek **[P-35s] [T-35s] [T-E9] [T-nos] [cry1Ac] [pat] [bar] . This combination allows the same coverage as the optimal one with 42% of expected cost saving (in comparison with 43% for the optimal combination)** As only T-nos and P35S are positive in this screening, we need to check for the presence of 87460, 88017, 89034, event 98140, GA21, LY038, MIR162, MIR604, Mon810, Mon863, NK603, 305423, 356043, RRS and BPS-CV127-9. Only RRS is positive.

Conclusion: 7 screening tests plus 15 event-specific tests = **total of 22 qualitative tests** (**49% saving in terms of tests**).

5plex combination:

[T-nos] [P35S] [P35S-pat] [CTP2-CP4EPSPS] [bar]. Combination covers all GMOs but 305423, BPS-CV127-9, Mon87701 and Mon87769.

As only T-nos and P-35S are positive in this screening, we need to check for the presence of 87460, 89034, Bt10, event 32, event 3272, event 98140, GA21, LY038, MIR162, MIR604, Mon810, Mon863, Bt63, 305423, 356043, RRS, BPS-CV127-9, Mon87701 and Mon87769 . Only RRS is positive.

Conclusion: 5 screening tests plus 19 event-specific tests = **total of 24 qualitative tests** (**44% saving in terms of tests**).

The combinations from GMOseek provide better GMO coverage than the 5plex combination and, in the case of the optimal combination, better cost efficiency. All combinations allow substantial savings when comparing to the current screening approach.

Sample 5

Background:

In the test sample 5, it was asked to use the double screening T-nos, P35S followed by identification of all possible authorized, tolerated and in pipeline maize lines in EU (including the UGM event 32 and Bt10). This resulted in 2 screening tests + 19 event-specific tests. **So, total of 21 qualitative tests.** The final conclusion was no presence of tested GMO while P-35S and T-nos showed positive signals. **This discrepancy was explained by the presence of traces of RRS in the sample. The screening strategy covers 17 of the possible 18 GMO events.**

Optimal combination proposed by GMOseek:

[P-35s] [T-35s] [T-nos]. Combination covers all 18 possible GMO events.

Theoretically saves 56% of the costs of analysis.

As P35S and T-nos are positive in this screening, we need to check for the presence of 14 of the 19 maize lines. All negative for these GM events.

Conclusion: 3 screening tests plus 14 event-specific tests = **total of 17 qualitative tests** (**19% saving in terms of tests**)

Note: the GMOseek algorithm then alerts that the results of screening phase are in contradiction with those of the identification phase.

5plex combination:

[T-nos] [P35S] [P35S-pat] [CTP2-CP4EPSPS] [bar]. Combination covers all GMOs.

As T-nos and P-35S are positive in this screening, we need to check for the presence of 10 of the 18 maize lines. All negative for these GM events.

Conclusion: 5 screening tests plus 13 event-specific tests = **total of 18 qualitative tests** (**17% saving in terms of tests**).

The combination from the 5plex approach provides a better coverage as the GMOseek combination but, with GMOseek, one less assay is needed to complete the analysis. Both combinations allow substantial savings when comparing to the current screening approach.

Sample 6

Background:

In the test sample 6, it was asked to use the double screening T-nos, P35S followed by identification of all possible authorized, tolerated and in pipeline maize lines in EU (including the UGM event 32 and Bt10). This resulted in 2 screening tests + 19 event-specific tests. The final conclusion was presence of Mon810. **So, total of 21 qualitative tests. The screening strategy covers 17 of the possible 18 GMO events.**

Note: using a simple DSS, only 8 event-specific would have been necessary instead of 19.

Optimal combination proposed by GMOseek:

[P-35s] [T-35s] [T-nos]. Combination covers all GMOs. Theoretically saves 56% of the costs of analysis.

As only P35S is positive in this screening, we need to check for the presence of 59122, event 98140, LY038, Mon810 and TC1507. Mon810 detected.

Conclusion: 3 screening tests plus 5 event-specific tests = **total of 8 qualitative tests** (62% saving in terms of tests, no saving if a DSS would have been used with the current screening)

5plex combination:

[T-nos] [P35S] [P35S-pat] [CTP2-CP4EPSPS] [bar]. Combination covers all GMOs.

As only P-35S is positive in this screening, we need to check for the presence of Mon810, event 32, event 98140, LY038 and 176. Only Mon810 is positive.

Conclusion: 5 screening tests plus 5 event-specific tests = **total of 10 qualitative tests** (**52% saving in terms of tests,** 2 more assays if a DSS would have been used with the current screening).

The combination from GMOseek provides same GMO coverage and better cost efficiency than the 5plex combination. Both combinations allow substantial savings when comparing to the current screening approach.

Sample 7

Background:

In the test sample 7, it was asked to use the double screening T-nos, P35S, followed by identification of all possible authorized, tolerated and in pipeline maize, soybean, and rice lines (including the UGM LL601, event 32, Bt10 and Bt63) in EU. This resulted in 2 screening tests + 31 event-specific tests. The final conclusion was no presence of the tested GMOs. **So, total of 33 qualitative tests.** It must be noted that for P35S, very high (39) Ct values were observed while T-nos gave negative result. **The screening strategy covers 24 of the possible 31 GMO events.**
Note: with such results, even if considering P35S positive, only the following targets should have been checked when using a DSS: 59122, 176, Event 32 (UGM), event 98140, LY038, Mon810, T25, Tc1507, LLRICE62, 305423, 356043, A2704-12 series, A5547127, BPS-CV127-9, Mon87701, Mon87769, and Mon89788. So 17 event-specific: **total of 19 qualitative tests (42% saved when comparing to what was done).**

Optimal combination proposed by GMOseek:

[P-35s] [T-35s] [T-E9] [T-nos] [T-pinII] [cry1Ac] [pat] [bar] [CTP2-CP4EPSPS]. Combination covers all GMOs but 305423, BPS-CV127-9.
Theoretically saves 44.85% of the costs of analysis.
With a positive P-35S result, we need to check for the presence of LY038, Mon810, 305423 and BPS-CV127-9.
Conclusion: 9 screening tests + 4 event specific= **total of 13 qualitative tests** (**60.5% saving in terms of tests, 31.5% of saving if a DSS would have been used with the current screening**)

5plex combination:

[T-nos] [P35S] [P35S-pat] [CTP2-CP4EPSPS] [bar]. Combination covers all GMOs but event 32, LY038, 305423, BPS-CV127-9, Mon87701 and Mon87769.
As only P35S would have been positive in this screening (but at the limit), we need to check for the presence of event 32, LY038, event 98140, Mon810, 305423, 350423, BPS-CV127-9, Mon87701 and Mon87769. All negative.
Conclusion: 5 screening tests plus 9 event-specific tests = **total of 14 qualitative tests** (**57.5% saving in terms of tests**)

GMOseek allows better GMO coverage and cost efficiency than the 5plex combination. Both combinations allow substantial savings when comparing to the current screening approach.

Cost comparison: “same strategy for all samples” (current approach at TestLab and 5plex approach) vs. “sample-centered GMO testing strategy” (GMOseek combinations)

Sample #	# assay old strategy	Cost old strategy	# assay 5plex	Cost 5plex	# assay GMOseek	Cost GMOseek
**sample 1**	40	939.02	23	578.96	16	430.7
**sample 2**	21	536.6	11	324.8	10	303.62
**sample 3**	33	790.76	18	473.06	17	451.88
**sample 4**	43	1002.56	24	600.14	20	515.42
**sample 5**	21	536.6	18	473.06	17	451.88
**sample 6**	21	536.6	10	303.62	8	261.26
**sample 7**	33	790.76	14	388.34	13	367.16

Comparison of the total analysis cost for the 7 above-described samples.
Cost were calculated using the linear function of the number of assays (numAssays) according to the equation $g(numAssays) = 21.18 \cdot numAssays + 91.82$. It is a simplification of the real situation with a relative absolute error of 3%.
