# Supplementary material for: GMOseek: a user friendly tool for optimized GMO testing
Source: BMC Bioinformatics. 2014 Aug 1;15(1):258. doi: 10.1186/1471-2105-15-258 (PMC4138379; doi:10.1186/1471-2105-15-258)
Supplement: Supplementary file 26 — Additional file 26: Raw results of the GMOseek robustness tests performed with equal frequencies. (PDF 219 KB) [file 12859_2013_6540_MOESM26_ESM.pdf]

Explanations

Below are given the combinations proposed by the GMOseek algorithm after simulations on subsets of the template EU GMO matrix. Description regarding these subsets is given in the section Near future 2 subsets of the deliverable D3/01.

Each simulation was interrupted after a long time of computation, meaning that the optimal solution is probably the last one found by the algorithm.

Each solution is provided as follows:

Time for completion (in milliseconds, ms)

Expected cost of the whole analysis (taking in account the screening phase and the identification phase costs)

(Expected cost of the whole analysis if only event-specific methods, cost saving in %)

Proposed combination (genetic elements to be targeted) (coverage of the solution: % coverage, ratio covered GM events/GM events in the matrix)

In the example below, the combination was provided in 203ms, expected cost is 1120,95 units while the cost using only event-specific method would be 1256.72. Therefore, expected savings on cost of analysis is 10.8%. The solution is a screening phase with P-35S only that covers 55% of the GM events to be analysed i.e. 30 of the 55 GM events in the matrix. The computation was interrupted after 37005000ms.

Example:

203ms Solution: 1120.95 (1256.72, 10.8%)

[P-35s] (55%, 30/55)

3705000ms

Interrupted!

Note that for the two last frequency levels, the best combination provide only low gain in terms of cost savings compared to the previously proposed combination(s) for the same GMO coverage.

First frequency level (1%)

32ms Solution: 742,22 (1044,92, 28,97%)

[P-35s] (56%, 25/45)

204ms Solution: 578,97 (1044,92, 44,59%)

[P-35s] [T-nos] (76%, 34/45)

579ms Solution: 492,9 (1044,92, 52,83%)

[P-35s] [T-E9] [T-nos] (87%, 39/45)

1063ms Solution: 452,99 (1044,92, 56,65%)

[P-35s] [T-E9] [T-nos] [pat] (91%, 41/45)

2547ms Solution: 452,3 (1044,92, 56,71%)

[P-35s] [P-Kti3] [P-ubiZM1] [T-E9] [T-nos] [pat] [CTP1] (96%, 43/45)

3516ms Solution: 449,41 (1044,92, 56,99%)

[P-35s] [P-Kti3] [P-ubiZM1] [T-E9] [T-nos] [m epsps] [pat] [CTP1] (98%, 44/45)

Covers all except BPS-CV127-9. No better combination after a long time

1972791ms

Interrupted!

Second frequency level (2%)

46ms Solution: 800,6 (1044,92, 23,38%)

[P-35s] (56%, 25/45)

312ms Solution: 644,77 (1044,92, 38,29%)

[P-35s] [T-nos] (76%, 34/45)

984ms Solution: 558,19 (1044,92, 46,58%)

[P-35s] [T-E9] [T-nos] (87%, 39/45)

2078ms Solution: 509,59 (1044,92, 51,23%)

[P-35s] [T-E9] [T-nos] [pat] (91%, 41/45)

3218ms Solution: 506,13 (1044,92, 51,56%)

[P-35s] [P-ubiZM1] [T-E9] [T-nos] [pat] (91%, 41/45)

4765ms Solution: 503,92 (1044,92, 51,77%)

[P-35s] [P-ubiZM1] [T-E9] [T-nos] [pat] [CTP1] (93%, 42/45)

6703ms Solution: 498,32 (1044,92, 52,31%)

[P-35s] [P-Kti3] [P-ubiZM1] [T-E9] [T-nos] [pat] [CTP1] (96%, 43/45)

9093ms Solution: 494,05 (1044,92, 52,72%)

[P-35s] [P-Kti3] [P-ubiZM1] [T-E9] [T-nos] [m epsps] [pat] [CTP1] (98%, 44/45)

Covers all except BPS-CV127-9

2760110ms

Interrupted!

Third frequency level (5%)

172ms Solution: 933,33 (1044,92, 10,68%)

[P-35s] (56%, 25/45)

1234ms Solution: 808,04 (1044,92, 22,67%)

[P-35s] [T-nos] (76%, 34/45)

4406ms Solution: 723,91 (1044,92, 30,72%)

[P-35s] [T-E9] [T-nos] (87%, 39/45)

9422ms Solution: 661,89 (1044,92, 36,66%)

[P-35s] [T-E9] [T-nos] [pat] (91%, 41/45)

15219ms Solution: 645,41 (1044,92, 38,23%)

[P-35s] [P-ubiZM1] [T-E9] [T-nos] [pat] (91%, 41/45)

23047ms Solution: 637,55 (1044,92, 38,99%)

[P-35s] [P-ubiZM1] [T-E9] [T-nos] [pat] [CTP1] (93%, 42/45)

33375ms Solution: 628,4 (1044,92, 39,86%)

[P-35s] [P-Kti3] [P-ubiZM1] [T-E9] [T-nos] [pat] [CTP1] (96%, 43/45)

46531ms Solution: 620,95 (1044,92, 40,57%)

[P-35s] [P-Kti3] [P-ubiZM1] [T-E9] [T-nos] [m epsps] [pat] [CTP1] (98%, 44/45)

60719ms Solution: 620,82 (1044,92, 40,59%)

[P-35s] [P-Kti3] [P-ubiZM1] [T-E9] [T-nos] [m epsps] [pat] [CTP1] [CTP2-CP4EPSPS] (98%, 44/45)

76485ms Solution: 599,89 (1044,92, 42,59%)

[P-35s] [P-Kti3] [P-ubiZM1] [T-E9] [T-nos] [m epsps] [nptII] [pat] [CTP1] [CTP2-CP4EPSPS] (98%, 44/45)

93204ms Solution: 593,91 (1044,92, 43,16%)

[P-35s] [P-Kti3] [P-ubiZM1] [T-E9] [T-nos] [m epsps] [nptII] [pat] [bar] [CTP1] [CTP2-CP4EPSPS] (98%, 44/45)

Covers all except BPS-CV127-9

1165445ms

Interrupted!

Fourth frequency level (10%)

8484ms Solution: 986,81 (1044,92, 5,56%)

[P-35s] [T-nos] (76%, 34/45)

41484ms Solution: 916,17 (1044,92, 12,32%)

[P-35s] [T-E9] [T-nos] (87%, 39/45)

100297ms Solution: 857,45 (1044,92, 17,94%)

[P-35s] [T-E9] [T-nos] [pat] (91%, 41/45)

171094ms Solution: 837,42 (1044,92, 19,86%)

[P-35s] [P-ubiZM1] [T-E9] [T-nos] [pat] (91%, 41/45)

277689ms Solution: 819,88 (1044,92, 21,54%)

[P-35s] [P-ubiZM1] [T-E9] [T-nos] [pat] [CTP1] (93%, 42/45)

430502ms Solution: 808,67 (1044,92, 22,61%)

[P-35s] [P-Kti3] [P-ubiZM1] [T-E9] [T-nos] [pat] [CTP1] (96%, 43/45)

637894ms Solution: 798,81 (1044,92, 23,55%)

[P-35s] [P-Kti3] [P-ubiZM1] [T-E9] [T-nos] [m epsps] [pat] [CTP1] (98%, 44/45)

863193ms Solution: 791,03 (1044,92, 24,3%)

[P-35s] [P-Kti3] [P-ubiZM1] [T-E9] [T-nos] [m epsps] [pat] [CTP1] [CTP2-CP4EPSPS] (98%, 44/45)

1117194ms Solution: 744,63 (1044,92, 28,74%)

[P-35s] [P-Kti3] [P-ubiZM1] [T-E9] [T-nos] [m epsps] [nptII] [pat] [CTP1] [CTP2-CP4EPSPS] (98%, 44/45)

1391743ms Solution: 726,25 (1044,92, 30,5%)

[P-35s] [P-Kti3] [P-ubiZM1] [T-E9] [T-nos] [m epsps] [nptII] [pat] [bar] [CTP1] [CTP2-CP4EPSPS] (98%, 44/45)

1683010ms Solution: 718,07 (1044,92, 31,28%)

[P-35s] [P-Kti3] [P-ubiZM1] [T-E9] [T-nos] [CryIaB / CryIac] [m epsps] [nptII] [pat] [bar] [CTP1] [CTP2-CP4EPSPS] (98%, 44/45)

Covers all except BPS-CV127-9

57340172ms Interrupted!
